# Serum Free Fatty Acid Changes Caused by High Expression of Stearoyl-CoA Desaturase 1 in Tumor Tissues Are Early Diagnostic Markers for Ovarian Cancer

**DOI:** 10.1158/2767-9764.CRC-23-0138

**Published:** 2023-09-13

**Authors:** Kanoko Katoh, Yuki Katoh, Akiko Kubo, Miho Iida, Yuji Ikeda, Takashi Iwata, Hiroshi Nishio, Masaki Sugawara, Daiki Kato, Makoto Suematsu, Shuichi Hirai, Kei Kawana

**Affiliations:** 1 Department of Obstetrics and Gynecology, Nihon University School of Medicine, Tokyo, Japan.; 2 Division of Anatomical Science, Department of Functional Morphology, Nihon University School of Medicine, Tokyo, Japan.; 3 Department of Obstetrics and Gynecology, Keio University School of Medicine, Tokyo, Japan.; 4 Department of Biochemistry, Keio University School of Medicine, Tokyo, Japan.; 5 Department of Preventive Medicine and Public Health, Keio University School of Medicine, Tokyo, Japan.; 6 Graduate School of Agricultural and Life Sciences, The University of Tokyo, Tokyo, Japan.; 7 WPI-Bio2Q Research Center and Central Institute for Experimental Animals, Kawasaki, Japan.

## Abstract

Ovarian cancer has a poor prognosis and is difficult to detect in early stages. Therefore, developing new diagnostic markers for early-stage ovarian cancer is critical. Here, we developed a diagnostic marker for early-stage ovarian cancer on the basis of fatty acid metabolism characteristics of cancer cells. The expression of various fatty acid metabolizing enzymes such as stearoyl-CoA desaturase 1 (SCD1) was altered in early-stage ovarian cancer tissue compared with that in normal ovarian tissue. Changes in the expression of fatty acid metabolizing enzymes, particularly SCD1, in cancer tissues were found to alter concentrations of multiple free fatty acids (FFA) in serum. We were the first to show that fatty acid metabolic characteristics in tissues are related to the FFA composition of serum. Surprisingly, patients with stage I/II ovarian cancer also showed significant changes in serum levels of eight FFAs, which can be early diagnostic markers. Finally, using statistical analysis, an optimal early diagnostic model combining oleic and arachidic acid levels, fatty acids associated with SCD1, was established and confirmed to have higher diagnostic power than CA125, regardless of histology. Thus, our newly developed diagnostic model using serum FFAs may be a powerful tool for the noninvasive early detection of ovarian cancer.

**Significance:**

Measurement of serum FFA levels by changes in the expression of fatty acid metabolizing enzymes in tumor tissue would allow early detection of ovarian cancer. In particular, the SCD1-associated FFAs, oleic and arachidic acid, would be powerful new screening tools for early-stage ovarian cancer.

## Introduction

Ovarian cancer is a tumor that develops in the abdominal cavity. Because pathologic diagnosis by biopsy is not possible and early detection is extremely difficult, approximately 70% of ovarian cancers are diagnosed at stage III or IV (1). For advanced stage cancers, multidisciplinary treatment with surgical resection and chemotherapy is performed; however, approximately 80% of tumors are refractory with a poor prognosis, with frequent recurrence (2). As a result, ovarian cancer has the second highest mortality rate among gynecologic cancers worldwide (3). Effective screening and medical checkup methods for early diagnosis of ovarian cancer have also not been established. CA125 is widely accepted as a serum biomarker protein for ovarian cancer in current clinical practice, but it is insufficient as an indicator for early-stage tumors (4, 5). Other studies reported that HE4, TFPI2, and epithelial cell adhesion molecule (EpCAM, CD326) are tumor markers (6–10), and recent studies have shown that miRNAs in peripheral blood are highly sensitive markers (11). However, those markers have yet to be used in clinical practice, and cure rates have not improved in the past 30 years (12). Consequently, there is an urgent need for new, highly sensitive, convenient, and less invasive biomarkers that can be used to achieve early detection of ovarian cancer and to develop cancer screening tests.

Cancer cells use fatty acids for survival. Saturated and monounsaturated fatty acids are essential to cancer cells because they sustain membrane biosynthesis during the rapid proliferation of cancer cells and provide an important energy source during conditions of metabolic stress (13). Fatty acids also act as signaling molecules. For example, palmitoleic acid and oleic acid activate Wnt signaling and enhance cancer malignancy by stabilizing Wnt protein and inhibiting Wnt degradation, respectively (14–16). Stearoyl-CoA desaturase 1 (SCD1), the enzyme that produces these fatty acids, is highly expressed in many cancers, including breast, prostate, renal, lung, and ovarian cancer, making SCD1 an attractive target for cancer therapy (17, 18). Our previous study showed that high expression of SCD1 in tumor tissue dramatically alters serum free fatty acid (FFA) levels in patients with colon and lung cancer (19). However, no studies have examined the utility of cancer cell–specific fatty acid metabolism or fatty acids themselves in the diagnosis of ovarian cancer, especially in the early stages.

In this study, we found that abnormalities in fatty acid metabolism in ovarian cancer tissues alter the composition of FFAs in serum. These fatty acid levels are attractive targets as early diagnostic markers for ovarian cancer.

## Materials and Methods

### Clinical Samples

Ovarian tumor tissue samples were collected from 32 Japanese patients with ovarian cancer at the time of surgery. Nine normal ovarian tissue sample was obtained by removing grossly normal ovarian parenchyma from patients with benign diseases such as uterine fibroids or uterine adenomyosis who wish to have their ovaries removed. Serum samples were obtained from 30 patients with stage I and II ovarian cancer, 11 patients with stage III and IV ovarian cancer, and 30 healthy donors (Table 1). Ovarian tumor tissue, normal ovarian tissue, and serum samples from patients with ovarian cancer were obtained at the Department of Gynecology and Obstetrics, Nihon University Hospital (Tokyo, Japan) between 2017 and 2022. Serum samples from healthy donors were collected at Nihon University and Keio University (Tokyo, Japan). All samples were stored at −150°C until use. Clinicopathologic information of patients was collected from clinical records and pathology reports. Patients with ovarian cancer were classified from stage I to stage IV according to the clinical staging system of the International Federation of Gynecology and Obstetrics. Serum samples were collected consecutively from consenting patients before removal of the tumor at the time of initial surgery. To avoid bias due to treatment, serum sample was collected prior to any treatment, and cases treated with neoadjuvant chemotherapy were excluded from sample collection. All samples used in this study are Japanese, and therefore have a high percentage of clear-cell carcinoma. This trend is similar to previous reports and does not mean that the cohort used in this study is particularly strange (20). We obtained informed consent from patients for the use of clinical materials for research purposes.

**TABLE 1:** Characteristics of patients from whom serum samples were obtained

Characteristics	Healthy donors (*n* = 30)	Patients with ovarian cancer (*n* = 41)
Age		
Median (range)	44 (31–55)	52 (33–80)
Tumor size, *mm*		
median (range)		102 (20–280)
Histopathologic subtypes, *n (%)*		
Clear cell		14 (34.1%)
Endometrioid		5 (12.2%)
Serous		10 (24.4%)
Mucinous		6 (14.6%)
Others		6 (14.6%)
Pathologic stage, *n (%)*		
I–II		30 (73.2%)
III–IV		11 (26.8%)
PLN metastasis		
Negative/Positive		38/3
PAN metastasis		
Negative/Positive		34/7
Ascites		
None/small/moderate/massive		30/3/3/5

Abbreviations: PAN: paraaortic lymph node; PLN: pelvic lymph node.

### Animals and Cell Culture

BALB/c nude mice (6 to 8 weeks old, CLEA Japan) were bred at the animal facilities of Keio University (Tokyo, Japan), in accordance with the guidelines for animal experimentation. Mice were maintained in a specific pathogen-free environment on a 12-hour light–dark cycle, with the dark cycle occurring from 8:00 P.M. to 8:00 A.M.

The OVCAR-3 human ovarian adenocarcinoma cell line was purchased from the ATCC and cultured in RPMI1640 (Thermo Fisher Scientific) containing 10% heat-inactivated FBS, 100 U/mL penicillin, and 100 μg/mL streptomycin at 37°C with 5% CO_2_ under 100% humidity. OVCAR-3 cells were recovered from liquid nitrogen and cultured for at least five passages before used for experiment. Cell line was authenticated by short tandem repeat profile analysis (Promega). OVCAR-3 cells were verified to be negative for *Mycoplasma* contamination using TaKaRa PCR Mycoplasma Detection Set prior to experiments. Overexpression of human SCD1 in OVCAR-3 cells was established by puromycin selection as described previously (19).

### qRT-PCR Analysis

Total RNA was extracted from ovarian cancer tissues using the RNeasy Mini Kit (Qiagen). TaqMan RT-PCR primers and probes for human acetyl-CoA carboxylase 1 (*ACC1*), fatty acid synthase (*FASN*), stearoyl-CoA desaturase 1 (*SCD1*), fatty acid desaturase 1 (*FADS1*), *FADS2*, and elongation of very long-chain fatty acids 1–6 (*ELOVL1–6*) were purchased from Integrated DNA Technologies. qRT-PCR was performed using ReverTra Ace qPCR RT Kit (TOYOBO). Gene expression was determined following the 2^−ΔCt^ method, in which ΔCt is the difference between the mean Ct value of triplicate measurements of the sample and the endogenous GAPDH control.

### Sample Preparation for Gas Chromatography–Mass Spectrometry

We added 0.3 mL of PBS containing internal standards (100 ng of margaric acid) was added to 20 μL of serum, and the sample was mixed with a vortex. FFAs were extracted using an ISOLUTE SLE+ column (Biotage) and dichloromethane. The lower organic region of the sample was collected and dried under nitrogen stream. The residue was dissolved in 5 μL of pyridine and 30 μL of the reagent BSTFA+TMCS (99:1; TS-38831, Thermo Fisher Scientific) for trimethylsilylation. The derivatization reaction was performed for 30 minutes at 40°C.

### Gas Chromatography–Mass Spectrometry Analyses

Gas chromatography–mass spectrometry (GC-MS) analysis was performed on a Shimadzu GC-MS QP2010 Ultra equipped with an AOC20i autoinjector and Rtx-5MS column (30 m, 0.25 mm, 0.25 μm df) in the 70 eV electron ionization mode. The heating program was as follows: 150°C for 1 minute, 20°C/minute to 250°C, 5°C/minute to 280°C, hold 5 minutes, then 20°C/minute to 330°C, and hold for 3 minutes. The carrier gas was helium with a constant flow speed of 42.0 cm/second. One microliter was injected in a 5:1 split ratio with an injector temperature of 250°C; the MS interface temperature was held at 280°C. Selected ion monitoring for quantification was performed by recording the ions at *m/z* 311.20 for palmitoleic acid–trimethylsilyl derivative, *m/z* 313.20 for palmitic acid–trimethylsilyl derivative, *m/z* 327.20 for margaric acid–trimethylsilyl derivative, *m/z* 339.20 for oleic acid–trimethylsilyl derivative, and *m/z* 341.20 for stearic acid–trimethylsilyl derivative. The 18 FFAs evaluated are summarized in Table 2.

**TABLE 2:** GC-MS analyses of serum FFA

Compound	Abbreviation	Target ion *m/z*	Rt^a^ (minutes)	LOD^b^ (ng/20 μL)	LOQ^c^ (ng/20 μL)
Palmitoleic acid	FA 16:1	311.2	5.75	0.13	0.32
Palmitic acid	FA 16:0	313.2	5.833	0.09	0.22
γ-linolenic acid	FA 18:3 γ	335.2	6.569	1.89	4.96
Stearidonic acid	FA 18:4	333.2	6.611	0.75	1.75
Linoleic acid	FA 18:2	337.2	6.652	0.04	0.08
α-linolenic acid	FA 18:3 α	335.2	6.698	0.19	0.48
Oleic acid	FA 18:1	339.2	6.669	0.09	0.24
Vaccenic acid	FA 18:1 A	339.2	6.703	0.09	0.24
Stearic acid	FA 18:0	341.2	6.79	0.08	0.21
Arachidic acid	FA 20:0	369.2	7.917	0.12	0.31
Arachidonic acid	FA 20:4	361.2	7.54	2.58	6.58
Eicosapentaenoic acid	FA 20:5	359.2	7.595	9.65	21.31
Dihomo-γ-linoleic acid	FA 20:3	363.2	7.657	0.15	0.3
Docosahexaenoic acid	FA 22:6	385.25	8.798	0.91	2.25
Adrenic acid	FA 22:4	389.25	8.842	4.35	8.93
Docosapentaenoic acid	FA 22:5	387.25	8.915	0.43	1.02
Nervonic acid	FA 24:1	423.3	10.745	0.03	0.07
Lignocenic acid	FA 24:0	425.3	10.938	0.009	0.02
Margaric acid	FA 17:0	327.2	6.293	I.S.	I.S.

^a^Retention time.

^b^Limit of detection.

^c^Limit of quantification.

### Flowcytometric Analysis

OVCAR-3 mock and OVCAR SCD1 O.E. tumor cells were stained by anti-SCD1 Ab (clone: CD.E10; ab19862, Abcam). The secondary antibody used was DyLight 488 goat anti-mouse IgG (H+L; ab96879, Abcam) at 1:500 dilution for 1 hour at 4°C and then washed three times with FACS buffer (2% FCS PBS).

### Tumor-bearing Animal Experiments

Nude mice were inoculated subcutaneously in the flank with 1 × 10^7^ OVCAR-3 mock cells and SCD1-overexpressing OVCAR-3 cells (*n* = 5/group). The tumor volume was calculated every 4 days using the formula: [length × (width)^2^]/2. Serum was collected 20 days after cancer cells implantation.

### Statistical Analysis

Unsupervised clustering and heat map generation with sorted datasets were performed using MetaboAnalyst 5.0. Comparisons between two groups were assessed using unpaired or paired (for matched comparisons) two-tailed Student *t* tests or nonparametric Mann–Whitney *U* tests. Multiple comparisons were assessed by one-way ANOVA, including Tukey or Bonferroni multiple comparisons tests. Pearson correlation coefficient was used to calculate the association between the SCD1 gene expression level in tumor and FFA ratio in serum. The diagnostic sensitivity, specificity, accuracy, and area under the ROC curve (AUC) were calculated for each FFA.

For the construction of an optimal diagnostic model for early-stage ovarian cancer, samples were randomly divided into discovery and validation sets using a 2:1 ratio stratified by malignancy status (control/early-stage ovarian cancer). The discovery set was used to select lipid markers and construct a model, and the validation set was used to validate it. Using the discovery set, multivariable analysis was conducted using the logistic regression model. Variables were first selected on the basis of univariate screening by comparing those of healthy controls and stage I/II ovarian cancer, which yielded to eight fatty acids with significant differences, followed by a combination of forward-selection and backward-elimination procedures. Correlations between serum concentrations of lipid markers were calculated using Spearman rank correlation method to assess for multicollinearity. The variable selection procedure was set to a threshold of 0.05 for inclusion and 0.05 for exclusion. The Akaike Information Criterion (AIC) was applied to determine the best model among the models. Diagnostic index (DI) score was calculated using the intercept and coefficients rounded to one decimal place. Finally, the performance of the DI was assessed in the validation dataset. Sensitivity analysis was performed by including late-stage cases. For comparison, a DI with CA125 values was also calculated using the discovery dataset. For each DI, the diagnostic sensitivity, specificity, accuracy, and AUC were calculated.

SAS software version 9.4 (SAS Institute), Prism software (version 9.0; GraphPad Software), and MetaboAnalyst 5.0 were used for data management and statistical analyses. Significance levels were set at *P* < 0.05 for all tests. Data are presented as mean ± SD.

### Ethics Statement

The study was conducted according to the guidelines of the Declaration of Helsinki and was approved by Nihon University Institutional Review Board (RK-170711-6, approved on August 25, 2017 and RK-200609-1, approved on September 9, 2020) and Keio University Institutional Review Board (20110159, approved on September 26, 2011 and 20130122 approved on August 7, 2013). We obtained written informed consent from patients for the use of clinical materials for research purposes. All animal studies were reviewed and approved by the Keio University Institutional Animal Care and Use Committee (approval number: 4062). Recombinant DNA experiments were reviewed and approved by Genetic Modification Safety Committee, Keio University School of Medicine (Tokyo, Japan; approval number: 25-014-13).

### Data Availability

All data relevant to the study are included in the article or uploaded as Supplementary Data.

## Results

### Expressions of Fatty Acid Metabolizing Enzymes are Altered in Ovarian Cancer Tissue

**FIGURE 1:**
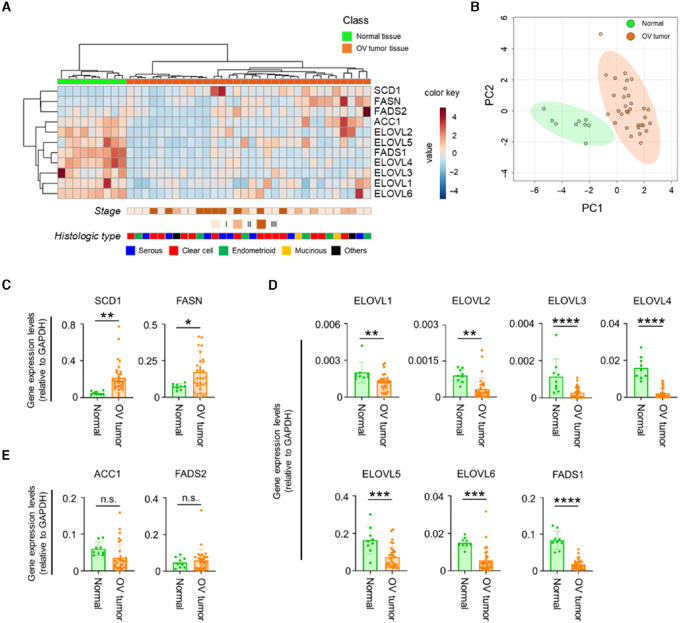
Gene expression of fatty acid metabolizing enzymes in ovarian tumor tissue and normal ovarian tissue. Gene expression of fatty acid metabolizing enzymes (ACC1, FASN, SCD1, FADS1, FADS2, and ELOVL1–6) in normal ovarian tissues (*n* = 9) and ovarian (OV) tumor tissues (*n* = 32) were measured by qRT-PCR. **A,** Heat map of the expression of 11 fatty acid metabolizing enzymes in each sample. **B,** Principal component analysis of the expression patterns of fatty acid metabolizing enzymes. **C–E,** Comparison of the expression levels of fatty acid metabolizing enzymes. Enzymes with higher expression in ovarian tumor tissue (**C**), enzymes with lower expression in ovarian tumor tissue (**D**), and enzymes with unchanged expression are shown (**E**). *, *P* < 0.05; **, *P* < 0.01; ***, *P* < 0.001; ****, *P* < 0.0001; n.s., not significant.

Previous studies have shown that many kinds of cancer tissues have specific metabolisms that differ from those of normal tissues (13). To determine whether fatty acid metabolism is altered in ovarian cancer tissue, we first evaluated the gene expression of fatty acid synthases (ACC1, FASN), fatty acid desaturases (SCD1, FADS1, FADS2) and fatty acid elongase 1–6 (ELOVL1–6) in ovarian tumor tissues and normal ovarian tissues. The results showed that the gene expression of several enzymes related to fatty acid metabolism, such as SCD1, FASN, and ELOVL, were altered in ovarian cancer tissues (Fig. 1A). The different expression of fatty acid metabolizing enzymes in ovarian cancer tissue compared with that in normal ovarian tissue was also confirmed by principal component analysis (Fig. 1B). SCD1 and FASN expressions were higher and ELOVL1–6 and FADS1 expressions were lower in ovarian cancer tissue, while ACC1 and FADS2 were unchanged (Fig. 1C–E). In addition, changes in expression of fatty acid metabolizing enzymes were independent of stage or histologic type (Supplementary Fig. S1 and S2). These results strongly suggest that fatty acid metabolism is changed in ovarian cancer tissue.

### The Composition of FFAs in Serum is Altered in Patients with Ovarian Cancer

**FIGURE 2:**
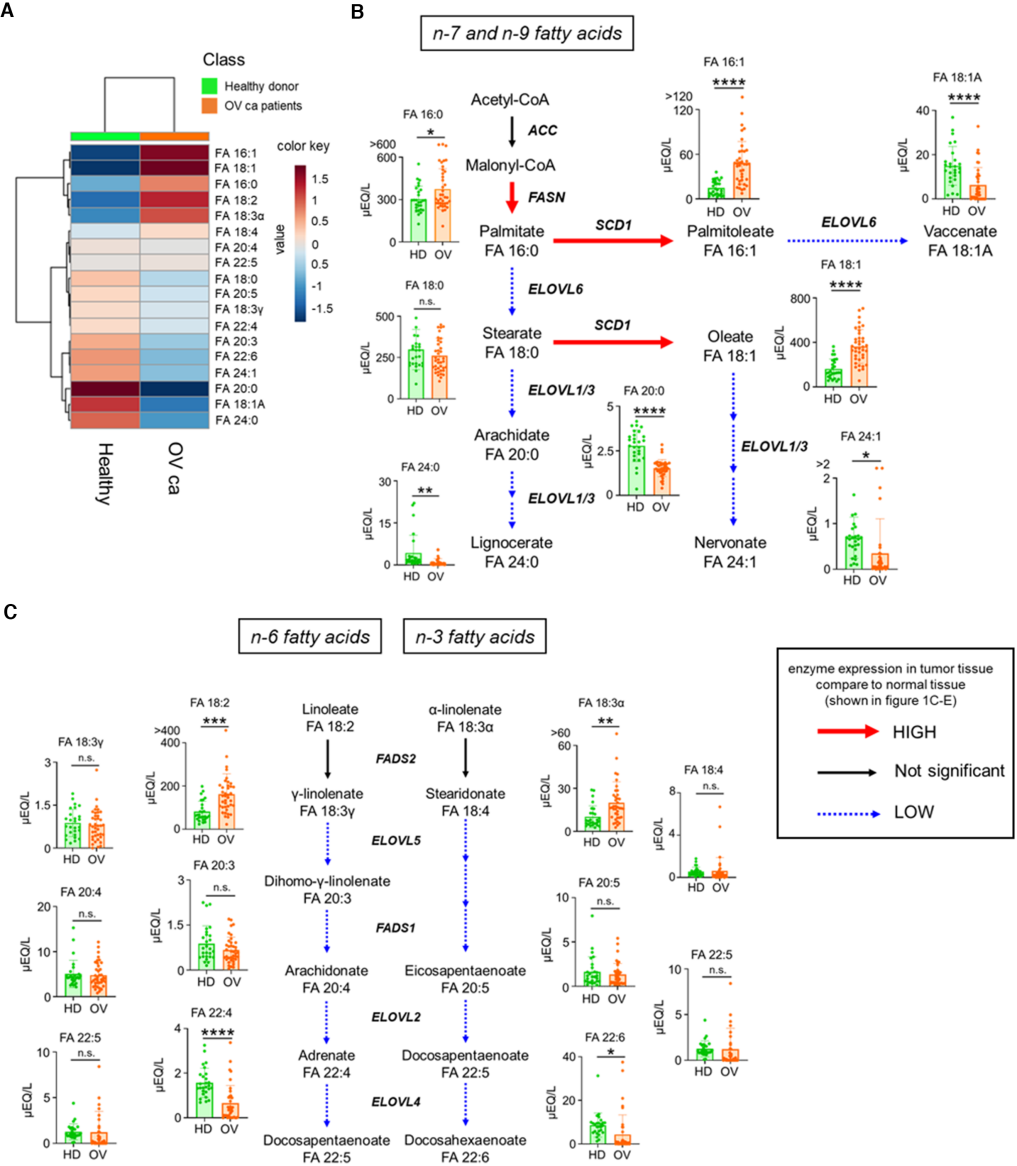
Serum FFA levels in patients with ovarian cancer and healthy donors. The levels of 18 FFAs (shown in Table 2) were measured by GC-MS in the serum of healthy donors (*n* = 30) and patients with stage I to IV ovarian cancer (*n* = 41). **A,** Heat map of average values for serum levels of FFAs. Comparison of serum FFA levels and map of n-7 and n-9 (**B**) and map of n-3 and n-6 (**C**) fatty acid metabolic pathways. The color of the arrows in the metabolic map indicates the higher or lower expression of mRNA encoding lipid-metabolizing enzymes in ovarian cancer tissue compared with that in normal tissue. *, *P* < 0.05; **, *P* < 0.01; ***, *P* < 0.001; ****, *P* < 0.0001; n.s., not significant; HD, healthy donor; OV, ovarian carcinoma patient.

We previously reported that the composition of FFAs in serum is altered by changes in the expression of fatty acid metabolizing enzymes in tumor tissues (19). Using the previously reported method, we performed quantitative analysis of 18 FFAs in serum samples from healthy controls and patients with ovarian cancer (Tables 1 and 2). Compared with healthy controls, ovarian cancer patients had higher levels of palmitic (FA16:0), palmitoleic (FA16:1), oleic (FA18:1), linoleic (FA18:2), and α-linoleic acids (FA18:3α) and lower levels of vaccenic (FA18:1A), arachidic (FA20:0), adrenic (FA22:4), docosahexaenoic (FA22:6), lignoceric (FA24:0), and nervonic acids (FA24:1; Fig. 2A). Notably, these changes in fatty acid levels correlated with changes in the expression of fatty acid metabolizing enzymes in the tissues shown in Fig. 1C–E (Fig. 2B and C). These results suggest that the serum FFA levels are altered in ovarian cancer tissues as a result of changes in fatty acid metabolic properties.

### Serum FFA Levels are Altered by Changes in the Expression of Fatty Acid Metabolizing Enzymes in Cancer Tissue

**FIGURE 3:**
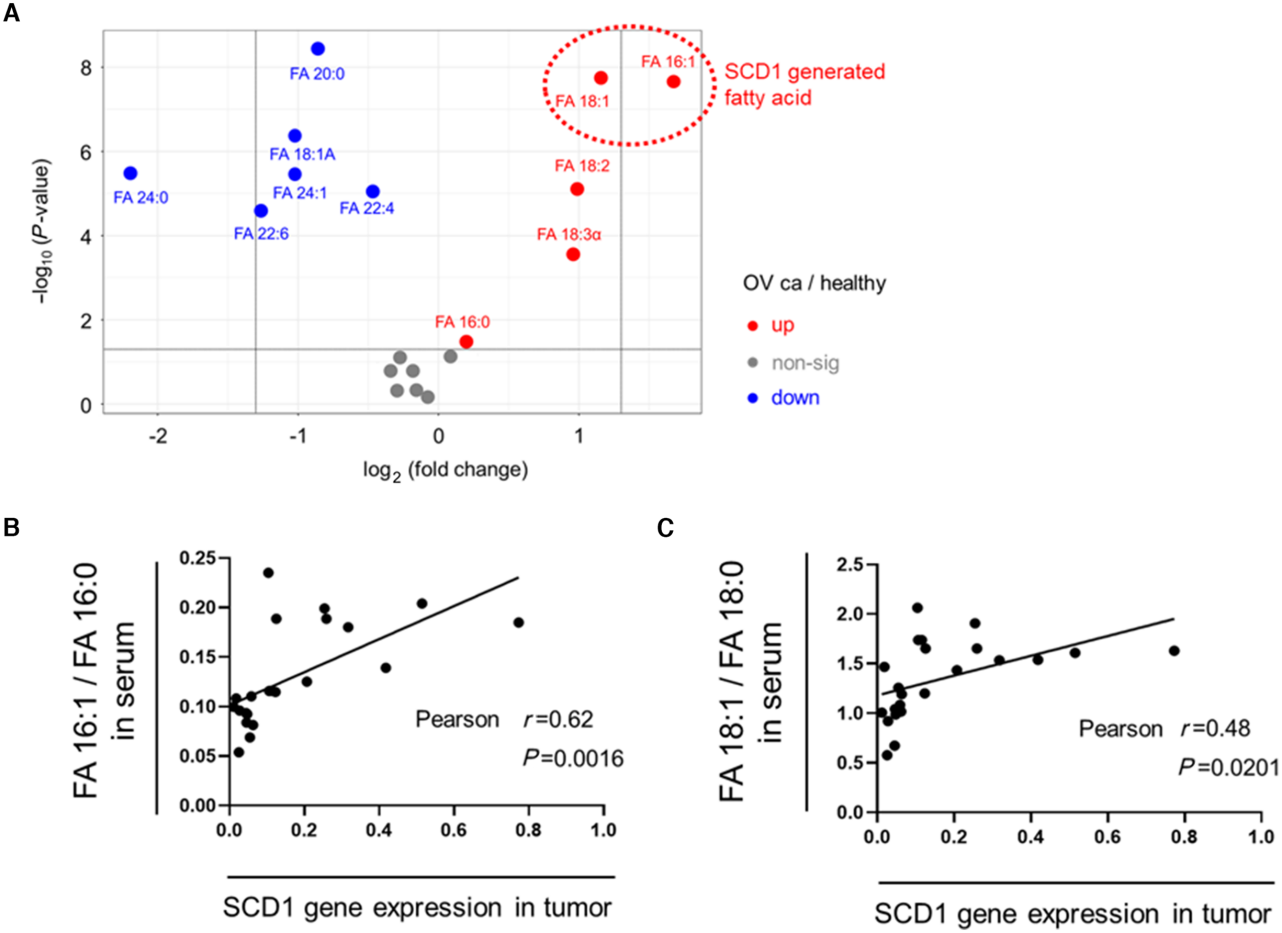
Correlation between SCD1 gene expression in tumor tissue and serum levels of SCD1-related FFAs in patients with ovarian cancer. **A,** Comparison of 18 FFAs in serum between patients with ovarian cancer and healthy donors, summarized in volcano plots. FFAs –log_10_ (*P* value) greater than 1.3 were considered significant. Correlation between SCD1 gene expression in ovarian tumor tissue and serum fatty acid ratios, FA16:1/FA16:0 (**B**) and FA18:1/18:0 (**C**), representing SCD1 activity, using paired samples (*n* = 23).

We next examined whether changes in fatty acid metabolism in ovarian cancer tissue influences serum FFA levels. We focused on the fact that the most highly altered fatty acids are palmitoleic acid and oleic acid produced by SCD1 (Fig. 3A) and analyzed the correlation between SCD1 expression in tumor tissue and fatty acid ratios, FA16:1/FA16:0, FA18:1/18:0, representing SCD1 activity in serum using paired samples (Fig. 3B and C). The results revealed a positive correlation between SCD1 expression and the fatty acid ratios, supporting our hypothesis.

**FIGURE 4:**
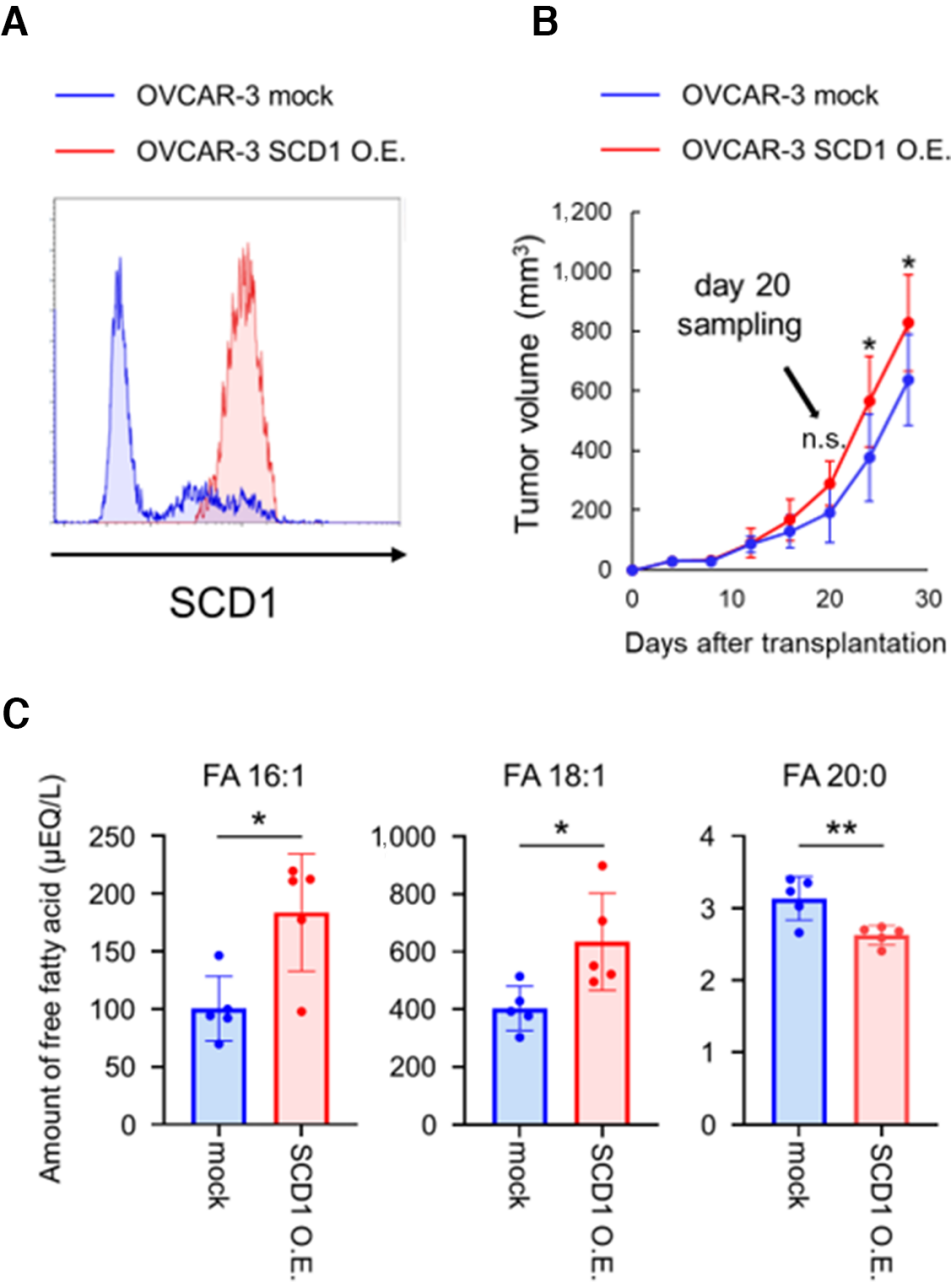
Relationship between SCD1 expression in tumor tissue and serum FFA levels in a tumor-bearing mouse model. Vector control OVCAR-3 (OVCAR-3 mock) or SCD1 overexpressing OVCAR-3 cells (OVCAR-3 SCD1 O.E.) were transplanted into nude mice. **A,** OVCAR-3 mock and OVCAR-3 SCD1 O.E. tumors were stained with anti-SCD1 mAb and analyzed using flow cytometry. **B,** Tumor growth curves (mean ± SD; *n* = 5). **C,** Serum levels of palmitoleic (FA16:1), oleic (FA18:1), and arachidic (FA 20:0) acids on day 20 after tumor cell transplantation (mean ± SD; *n* = 5). *, *P* < 0.05; **, *P* < 0.01; n.s., not significant.

To further confirm whether high expression of SCD1 in tumor tissues alters fatty acids in the serum, SCD1 was overexpressed in OVCAR-3, a human ovarian cancer cell line with low basal SCD1 expression, and transplanted into nude mice (Fig. 4A). The SCD1 overexpression group showed markedly increased tumor volume at 25 and 30 days after transplantation (Fig. 4B). To rule out the possibility that changes in serum fatty acid levels were dependent on tumor volume, serum samples were collected on day 20, when there was no difference in tumor volume between the SCD1 overexpression and control group. The levels of palmitoleic acid and oleic acid, SCD1 generated fatty acids, were markedly higher in the serum of the tumor-bearing mice implanted with SCD1-overexpressing cancer cells (Fig. 4C). Conversely, serum concentrations of arachidic acid, which like oleic acid is produced using stearic acid as a substrate, were markedly lower due to substrate depletion (Fig. 4C). These results suggest a parallel change in SCD1 expression and activity in tumor tissue and levels of FFAs in serum.

### Measurement of Serum FFA Levels Enables Early Diagnosis of Ovarian Cancer

To determine whether serum FFA levels can be used for cancer diagnosis, we performed ROC analysis on 11 FFAs that showed significant differences in serum levels in Fig. 2A–C. The results showed that 10 FFAs except palmitic acid (FA16:0) had AUC values higher than 0.7 and had power as diagnostic markers (Supplementary Fig. S3A and B).

**FIGURE 5:**
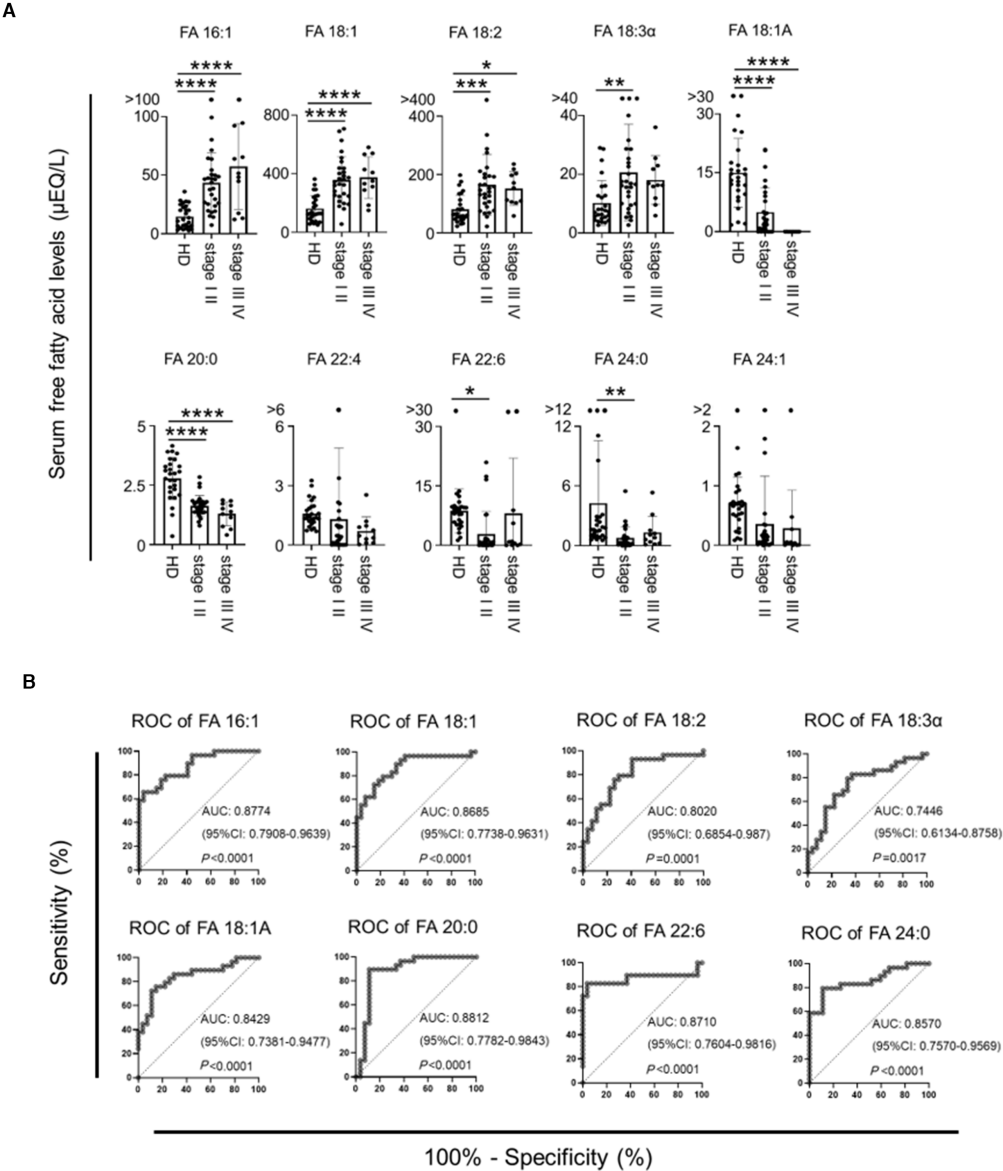
Serum FFA levels are useful as early diagnostic markers for ovarian cancer. **A,** Comparison of serum FFA levels in healthy donors (HD; *n* = 30) and patients with stage I/II (*n* = 30) and stage III/IV (*n* = 11) ovarian cancer. **B,** ROC curves for detecting patients with early-stage ovarian cancer using serum FFAs from A. *, *P* < 0.05; **, *P* < 0.01; ***, *P* < 0.001; ****, *P* < 0.0001.

Early detection of stage I and II ovarian cancer is essential to improve the prognosis of patients with ovarian cancer. Therefore, we next examined whether the 10 fatty acids could discriminate between patients with stage I/II ovarian cancer and healthy individuals. Compared with healthy controls, patients with stage I/II ovarian cancer had higher concentrations of FA 16:1, FA18:1, FA18:2, and FA18:3α and lower concentrations of FA18:1A, FA20:0, FA22:6, and FA24:0 (Fig. 5A). In addition, there was no correlation between changes in serum FFA concentrations of these eight species and the histologic type of ovarian cancer (Supplementary Fig. S4). When the diagnostic abilities of these eight fatty acids for early-stage disease were evaluated by ROC analysis, all eight showed a high diagnostic value (Fig. 5B). In addition, the levels of these eight FFAs were independent of the patient's body mass index (BMI; Supplementary Fig. S5). These results strongly suggest that specific FFA levels in serum may serve as diagnostic markers of early-stage ovarian cancer, regardless of histologic type.

### The Combination of FA18:1 and FA20:0 Detects Patients with Stage I/II Ovarian Cancer More Accurately Than CA125

**FIGURE 6:**
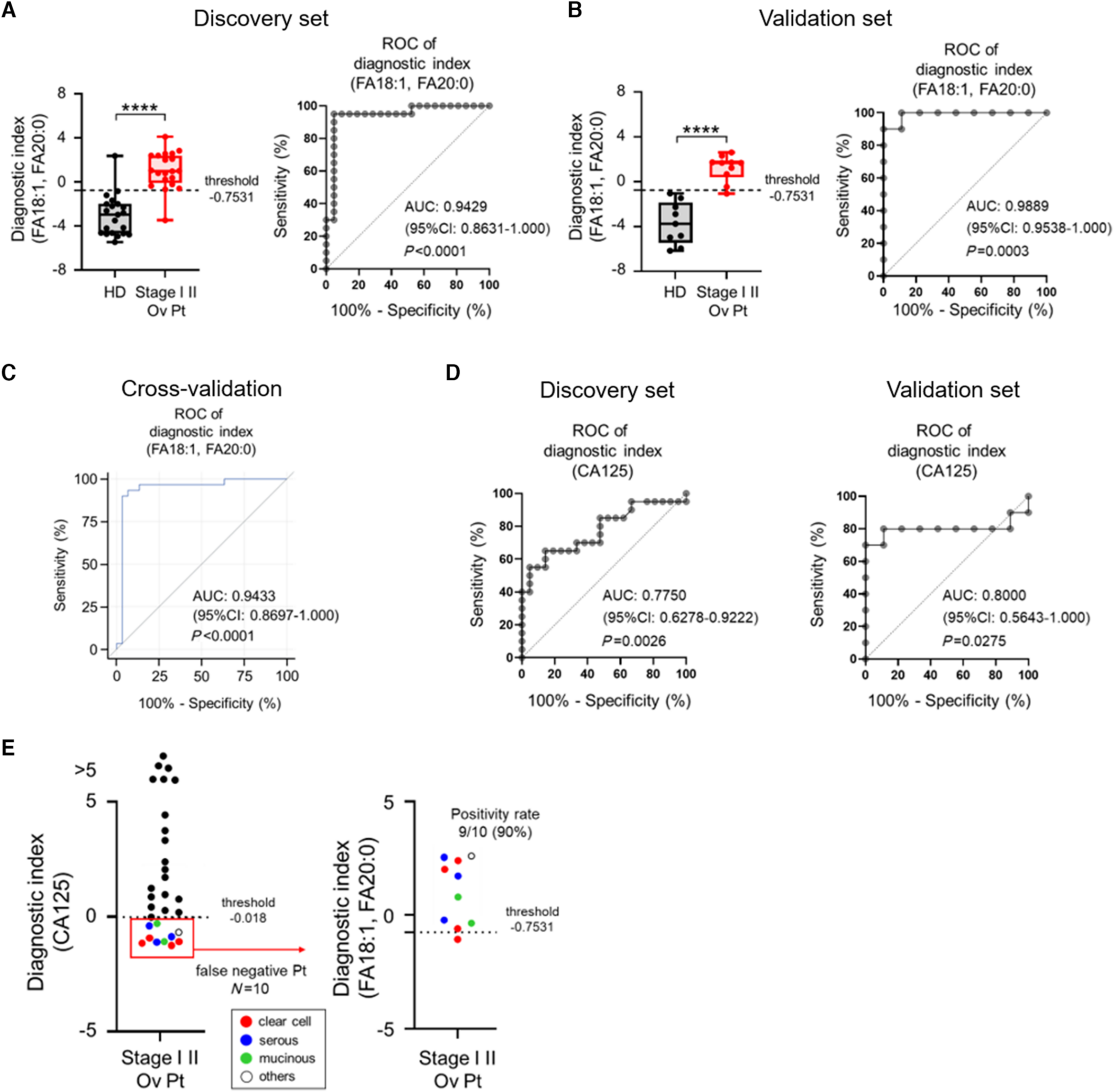
Development of the stage I/II ovarian cancer detection model using serum FFAs levels. Diagnostic performance of the diagnostic index using FA18:1 and FA20:0 in the discovery (**A**) and validation (**B**) sets. Left: Comparison between healthy donor (HD) and patients with stage I/II ovarian cancer (Ov Pt). Right: ROC analysis. Threshold was set by calculating Youden index by ROC analysis using the discovery set. **C,** ROC analysis with cross-validation method applied to the complete dataset. **D,** Diagnostic performance of diagnostic index using CA125 on the discovery (left) and validation (right) sets. **E,** Test performance of DI (CA125) and DI (FA18:1, FA20:0) combination. Left: Patients with stage I/II ovarian cancer (*n* = 30) were diagnosed using DI (CA125) values. Black dots indicate true positive and red (clear cell), blue (serous), green (mucinous), and white (others) dots indicate false negative. Right: Patients with a false negative diagnosis by DI (CA125; *n* = 10) were successfully diagnosed using DI (FA18:1, FA20:0). ****, *P* < 0.0001.

While the eight fatty acids identified in Fig. 5B showed sufficient power as early diagnostic markers on their own, there were still false negative and false positive cases. Therefore, we examined whether the combination of fatty acids could improve their diagnostic ability. First, using the discovery dataset (Supplementary Table S1), a multivariable logistic regression model was performed using the eight markers. A maximum of two explanatory variables was estimated to be included in the final model because there were 20 early-stage cases and 21 controls. With a combination of forward-selection and backward-elimination procedures, a combination of two markers associated with SCD1, FA18:1 and FA20:0, were identified to be statistically effective in distinguishing patients with ovarian cancer. The Spearman rank correlation coefficient between serum concentrations of FA18:1 and FA20:0 was −0.399, indicating less concern for multicollinearity. A DI formula was created using intercepts and coefficients rounded to one decimal place [DI = 0.9 + 0.01 × (FA18:1) + (–2.1) × FA20:0]. ROC analysis using DI with these two markers resulted in an AUC of 0.943 (Fig. 6A). The diagnostic performance of the model was assessed in the validation set (Supplementary Table S1), revealing that the model was very accurate, with an AUC of 0.989 (Fig. 6B). A cross-validation method was also applied to the complete dataset to assess the validity of the model, which yielded an AUC of 0.9433 (Fig. 6C). These results suggest the importance of combining various FFA markers in serum to improve diagnostic accuracy. Furthermore, the DI using CA125 values [DI (CA125)] showed AUC = 0.775 and AUC = 0.800 for the discovery and validation sets, respectively, indicating that the DI scoring system using FA18:1 and FA20:0 concentrations [DI (FA18:1, FA20:0)] demonstrated high predictive ability (Fig. 6D; Supplementary Table S2).

We also explored possibility of combining DI (CA125) with DI (FA18:1, FA20:0). The results showed that in the complete data set, 10 of 30 patients were false negative when diagnosed using CA125 (Fig. 6E, left). In patients who were subsequently false negative for CA125, diagnosis using DI (FA18:1, FA20:0) was positive in 9 of 10 patients (90%), and this result was independent of histopathologic subtypes including clear cell, serous, mucinous, and endometrioid (Fig. 6E, right). These results indicate that DI (FA18:1, FA20:0), using SCD1-related fatty acid levels, may represent an effective early diagnostic tool for ovarian cancer in clinical practice.

## Discussion

When ovarian cancer is detected at an early stage, the 5-year survival rate is 90% if the cancer is confined to the ovaries (stage I) or 70% if the cancer is confined to the pelvic region (stage II), and a complete cure is expected (21). However, most ovarian cancers are diagnosed at stages III and IV, and the 5-year survival rate is less than 30% (22, 23). Therefore, identifying new diagnostic markers for the early diagnosis of ovarian cancer is critical. Recent research has focused on changes in various metabolic characteristics of cancer cells, which are used to stratify patients with cancer. In this study, we demonstrated that the expressions of various fatty acid metabolizing enzymes are altered in ovarian cancer tissue compared with those in normal ovarian tissue, and the altered expression of metabolic enzymes in the tissue dramatically alters the FFA composition in serum. Furthermore, several of the altered FFAs clearly distinguished patients with ovarian cancer from healthy controls, particularly FA16:1, FA18:1, FA18:2, FA18:3α, FA18:1A, FA20:0, FA22:6, and FA24:0. These showed the ability to detect patients with stage I/II ovarian cancer with high accuracy and may serve as early diagnostic markers. Finally, the DI using FA18:1 and FA20:0 levels, fatty acids associated with SCD1, showed improved diagnostic accuracy over fatty acids alone. Furthermore, when used in combination with CA125, it was able to detect patients with stage I and II ovarian cancer with high accuracy, regardless of histology.

Most cancer cells express high levels of FASN and SCD1 and have increased de novo fatty acid synthesis compared with normal cells (17, 18, 24). Our results demonstrate that the expressions of FASN and SCD1 are also elevated in ovarian cancer tissues. Notably, serum levels of FA16:0, FA16:1, and FA18:1, metabolites of these enzymes, were also elevated. FASN is a key enzyme in *de novo* lipogenesis and supports both anabolic metabolism and oncogenic signaling (13). SCD1 is important in tumorigenesis because it not only maintains the structure and fluidity of the cell membrane and produces FA16:1 and FA18:1, fatty acids essential for cell proliferation, but it also regulates the intracellular pool of unsaturated fatty acids that later serve as building blocks for phosphoglycerides, phosphoinositides, eicosanoids, and sphingolipids (13, 25). As a result of these findings, the development of cancer therapies targeting these enzymes has intensified (26–30).

Recent studies reported that not only metabolic enzymes but also the products, fatty acids themselves, are involved in malignant transformation, with roles in cancer formation and invasion (13). For example, FA16:0, a fatty acid synthesized by FASN, promotes tumor metastasis in human oral cancer and human melanoma (31). Furthermore, FA16:0 is an important fatty acid for the palmitoylation of cysteine residues (S-palmitoylation) on cellular proteins (32). S-palmitoylation is known to play an important role in physiologic and pathologic processes in human cancer cells by affecting protein anchoring to the membrane, transport, interaction, and degradation (33). For example, cancer-related proteins such as EZH2 and c-Met, as well as the glucose transporter GLUT1, have been reported to be S-palmitoylated and stabilized to promote glioblastoma cell growth (34–36). In addition, it has also been recently reported that S-palmitoylation has been linked to immune escape of breast cancer cells by maintaining PD-L1 protein stability and cell surface distribution (37). Other than FA16:0, FA16:1 and FA18:1 contribute to the malignant transformation of cancer by upregulating oncogenic signaling such as Wnt/β-catenin signaling, thereby creating an immunosuppressive cancer microenvironment (14–16, 19). We also showed elevated levels of FA18:2 and FA18:3α, the first fatty acids in the ω3 and ω6 fatty acid metabolic pathways, in ovarian cancer tissues. FA18:2 is metabolized to arachidonic acid and subsequently to some prostaglandins, leukotrienes, and thromboxanes, which are involved in inflammatory responses (38). FA18:3α has been identified as a fatty acid with anti-inflammatory properties, but a recent meta-analysis of 1,197,564 individuals showed that dietary FA18:3α intake was associated with a slightly increased risk of cancer death (39).

Our findings also showed that levels of four fatty acids, FA18:1A, FA20:0, FA22:6, and FA24:0, were lower in patients with ovarian cancer compared with those in healthy controls. Previous reports have indicated that these FFAs are inhibitors of cancer cell growth. FA18:1A was reported to inhibit mammary gland premalignant lesions and induce apoptosis of human nasopharyngeal carcinoma cells (40, 41). FA20:0 enhances ferroptosis susceptibility in gastric cancer (42). Other reports indicate that FA22:6 exerts antitumor effects in pancreatic cancer cells via induction of oxidative stress and inhibition of β-catenin accumulation (43, 44). These findings indicate that ovarian cancer cells may inhibit the production of fatty acids that interfere with their survival.

Correlation analysis using paired clinical samples of ovarian cancer tissue and serum from a tumor-bearing mouse model and patients with ovarian cancer revealed that changes in the expression of fatty acid metabolizing enzymes in ovarian cancer tissue were associated with changes in serum concentrations of FFAs. These findings suggested that ovarian cancer cells establish a cancer microenvironment suitable for survival through specific fatty acid metabolism, and the release of pooled FFAs into the serum may alter the serum composition of fatty acids.

Ultrasound imaging and blood tests, particularly CA125, have been used to detect ovarian cancer in its early stages (45–48). CA125 is expressed in human epithelial ovarian cancer and normal endometrium, lung, and cornea; it is cleaved and shed into the blood and can be measured by immunoassay (49–51). CA125 was detected in 90% of patients with stage III–IV ovarian cancer, but only in 60% of patients with stage I–II ovarian cancer (50), similar to our results, and is known to be a poor early diagnostic marker, especially in mucinous and clear cell carcinomas, because of its low positive rate (52, 53). Because of these issues, recent studies have investigated serum markers such as HE4 (6), TFPI2 (9), circulating tumor DNA (54, 55), DNA methylation (56), and circulating miRNA (11) as complementary markers to CA125. Circulating miRNAs have been reported to have high diagnostic performance in several cohorts and are being developed as independent markers for the early diagnosis of ovarian cancer independent of CA125 (11, 57, 58). However, it has not yet led to a dramatic improvement in prognosis with an increased cure rate (12). In the current study, we analyzed FFAs in serum from ovarian cancer cases from the perspective of exploring fatty acid metabolism in cancer and identified eight FFAs, FA16:1, FA18:1, FA18:2, FA18:3α, FA18:1A, FA20:0, FA22:6, and FA24:0, as potential early diagnostic markers for ovarian cancer. Studies have reported elevated FA16:1 and FA18:1 levels in patients with breast cancer of all stages (59), and studies of patients with early-stage colorectal, gastric, and non–small cell lung cancer have reported low levels of FA22:6 (60–62). These reports support our findings that it is possible to stratify patients with cancer by serum FFA levels.

We previously reported that serum FFA levels partially correlate with tissue metabolic enzyme expression in patients with colorectal and lung cancer (19). Thus, early diagnosis of cancer by serum FFA levels may be possible not only in ovarian cancer but also in other cancer types. These findings also suggest that there may be fatty acids that are commonly altered in various cancer types. Therefore, to improve specificity as a diagnostic marker, multiple fatty acids in combination should be evaluated, rather than individual fatty acids. In our study, the combination of FA18:1 and FA20:0 was identified as the best diagnostic model for ovarian cancer. The DI using FA18:1 and FA20:0 levels [DI (FA18:1, FA20:0)] detected 95% and 100% of patients with stage I/II ovarian cancer in the discovery and validation sets, respectively, despite the small cohort size. The index showed diagnostic performance comparable with that of circulating miRNAs, which are known to be highly accurate markers (57). Furthermore, 90% patients in this cohort who were false negative by CA125 were detectable by DI (FA18:1, FA20:0; Fig. 6E). In particular, patients with clear cell carcinoma, which is difficult to detect with CA125 (52, 53), were detected with high sensitivity. This suggests that our DI (FA18:1, FA20:0) is not only useful as an independent early diagnostic marker but also complementary to CA125 and can coexist with existing diagnostic markers.

This study has some limitations due to the small sample size. First, for the application of these biomarkers as early diagnostic markers in clinical practice, their specificity needs to be verified, it will be necessary to construct an optimal model by investigating comparisons with benign diseases such as endometriosis and other cancer types and associations with factors such as menstruation and pregnancy. In addition, combination with other diagnostic markers, such as CA125 and miRNA (11), should be considered to improve specificity. Second, the cohort is different from United States or Europe because all patients enrolled in this study are Japanese who underwent surgery as first-line treatment. Although the early diagnosis model using serum FFAs was independent of stage and histology, it needs additional investigation in a large cohort of multicenter clinical samples, including United States or Europe. Finally, the effect of diet should be considered. Although little effect of BMI or short-term fasting was observed (Supplementary Fig. S5 and S6), long-term diet will have a significant impact on serum FFA levels. Therefore, it will be necessary to establish the baseline of FFAs in the serum of the normal population, taking into account dietary culture.

In summary, our comprehensive fatty acid metabolic analysis of tissue and serum from patients with ovarian cancer identified a combination of serum FFAs that is promising for the early detection of ovarian cancer. In clinical practice, noninvasive liquid biopsy is critically needed because repeated tissue biopsies are almost impossible to obtain. The diagnostic model we developed, together with the existing tumor marker CA125 and miRNA, which is currently under development, will be a powerful tool for the early diagnosis of ovarian cancer.

## Supplementary Material

Figure S1 Supplemental figure S1. Expression of fatty acid metabolizing enzymes in ovarian cancer tissue among clinical stages, related to Figure 1. Comparison of gene expression of 11 fatty acid metabolizing enzymes in cancer tissues among stage I (n=17), II (n=6), and III (n=9). n.s., not significant.

Figure S2 Supplemental figure S2. Expression of fatty acid metabolizing enzymes in ovarian cancer tissue among histologic types, related to Figure 1. Comparison of gene expression of 11 fatty acid metabolizing enzymes in cancer tissues among serous (n=8), clear cell (n=14) and endometrioid + mucinous + others (E+M+O) (n=10). n.s., not significant.

Figure S3 Supplemental figure S3. Diagnostic potential of levels of 11 free fatty acids in patients with all stages of ovarian cancer. ROC analysis of serum free fatty acids with increased (A) and decreased (B) values in patients with all stages of ovarian cancer compared with healthy controls.

Figure S4 Supplemental figure S4. Serum free fatty acid levels in stage I and II ovarian cancer patients among histologic types, related to Figure 5. Comparison of serum free fatty acid levels in patients with stage I/II ovarian cancer patients of various histologic types (clear cell (n=10), serous (n=7), endometrioid (n=3), mucinous (n=6), others (n=4)). n.s., not significant.

Figure S5 Supplemental figure S5. Correlation between serum free fatty acid levels and BMI in patients with early-stage ovarian cancer. Analysis of the correlation between serum free fatty acid levels and BMI with the ability to diagnose stage I and II ovarian cancer patients, shown in Figure 5A and B.

Figure S6 Supplemental figure S6. Effect of short-term diet on serum free fatty acid levels. Serum was collected from healthy donors (n=7) pre and 2 hours post diet, and free fatty acids were measured. Gray lines represent individual values and black lines represent average values. n.s., not significant.

Table S1 Supplemental table S1. Characteristics of participants in the discovery set and the validation set.

Table S2 Supplemental table S2. Characteristics of DI (FA18:1, FA20:0) and DI (CA125) in the discovery set and validation set. Threshold was set by calculating Youden’s index by ROC analysis using the discovery set. DI (FA18:1, FA20:0): -0.7531, DI (CA125): -0.018.
