# Supplementary material for: Serum Free Fatty Acid Changes Caused by High Expression of Stearoyl-CoA Desaturase 1 in Tumor Tissues Are Early Diagnostic Markers for Ovarian Cancer
Source: Cancer Res Commun. 2023 Sep 13;3(9):1840–52. doi: 10.1158/2767-9764.CRC-23-0138 (PMC10498943; doi:10.1158/2767-9764.CRC-23-0138)
Supplement: Figure S1 — Supplemental figure S1. Expression of fatty acid metabolizing enzymes in ovarian cancer tissue among clinical stages, related to Figure 1. Comparison of gene expression of 11 fatty acid metabolizing enzymes in cancer tissues among stage I (n=17), II (n=6), and III (n=9). n.s., not significant. [file crc-23-0138-s01.docx]

**
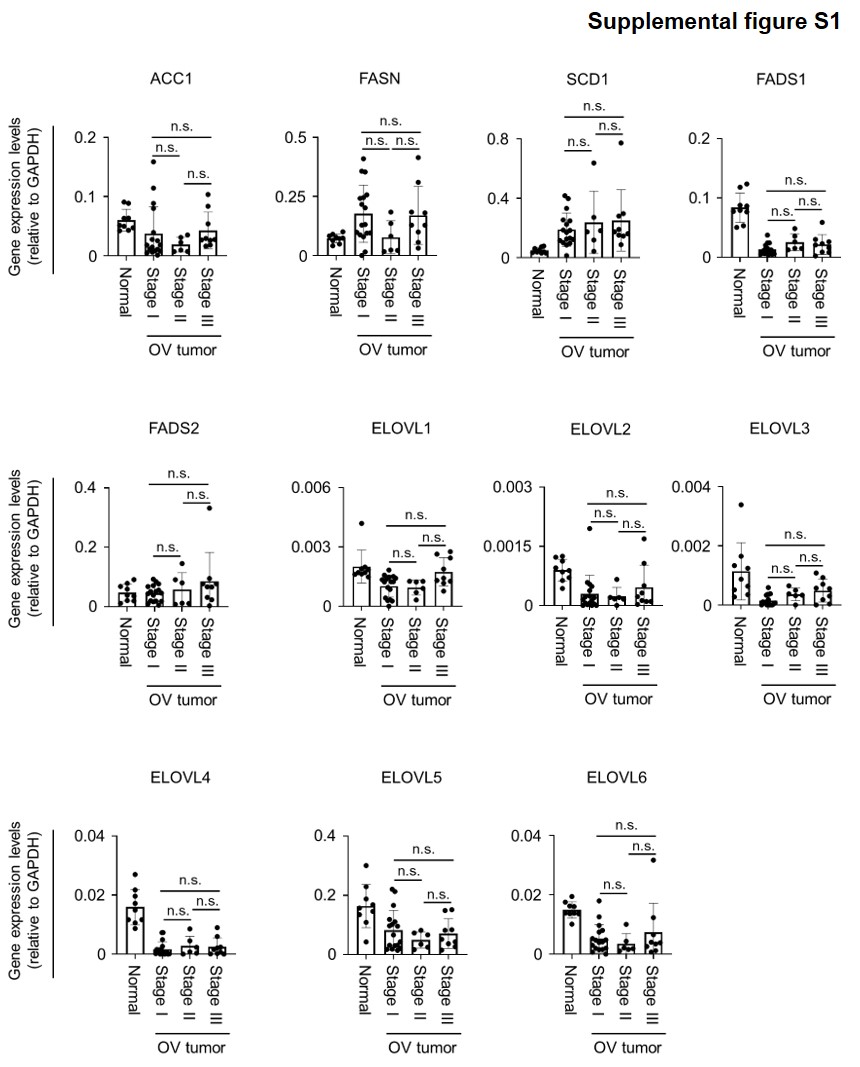
**

**Supplemental figure S1. Expression of fatty acid metabolizing enzymes in ovarian cancer tissue among clinical stages, related to Figure 1.** Comparison of gene expression of 11 fatty acid metabolizing enzymes in cancer tissues among stage I (n=17), II (n=6), and III (n=9). n.s., not significant.
