# Supplementary material for: Serum Free Fatty Acid Changes Caused by High Expression of Stearoyl-CoA Desaturase 1 in Tumor Tissues Are Early Diagnostic Markers for Ovarian Cancer
Source: Cancer Res Commun. 2023 Sep 13;3(9):1840–52. doi: 10.1158/2767-9764.CRC-23-0138 (PMC10498943; doi:10.1158/2767-9764.CRC-23-0138)
Supplement: Figure S2 — Supplemental figure S2. Expression of fatty acid metabolizing enzymes in ovarian cancer tissue among histologic types, related to Figure 1. Comparison of gene expression of 11 fatty acid metabolizing enzymes in cancer tissues among serous (n=8), clear cell (n=14) and endometrioid + mucinous + others (E+M+O) (n=10). n.s., not significant. [file crc-23-0138-s02.docx]

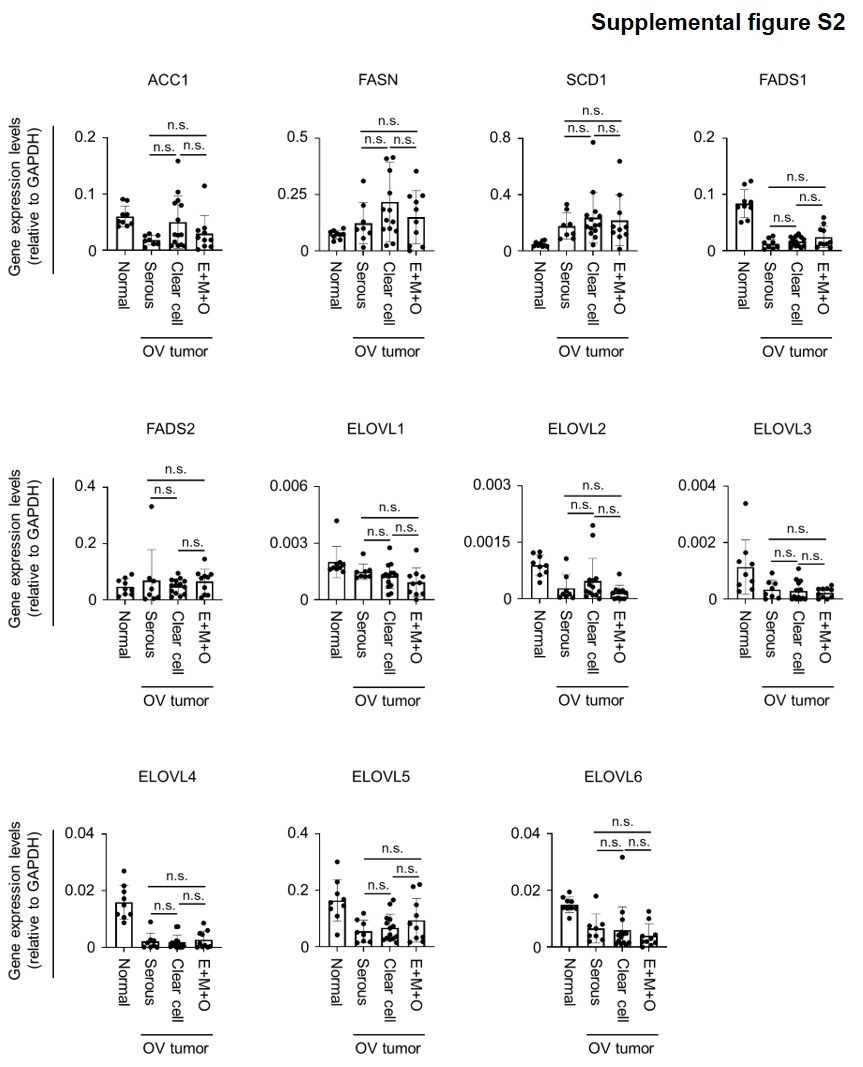


**Supplemental figure S2. Expression of fatty acid metabolizing enzymes in ovarian cancer tissue among histologic types, related to Figure 1.** Comparison of gene expression of 11 fatty acid metabolizing enzymes in cancer tissues among serous (n=8), clear cell (n=14) and endometrioid + mucinous + others (E+M+O) (n=10). n.s., not significant.
