# Supplementary material for: Serum Free Fatty Acid Changes Caused by High Expression of Stearoyl-CoA Desaturase 1 in Tumor Tissues Are Early Diagnostic Markers for Ovarian Cancer
Source: Cancer Res Commun. 2023 Sep 13;3(9):1840–52. doi: 10.1158/2767-9764.CRC-23-0138 (PMC10498943; doi:10.1158/2767-9764.CRC-23-0138)
Supplement: Figure S3 — Supplemental figure S3. Diagnostic potential of levels of 11 free fatty acids in patients with all stages of ovarian cancer. ROC analysis of serum free fatty acids with increased (A) and decreased (B) values in patients with all stages of ovarian cancer compared with healthy controls. [file crc-23-0138-s03.docx]

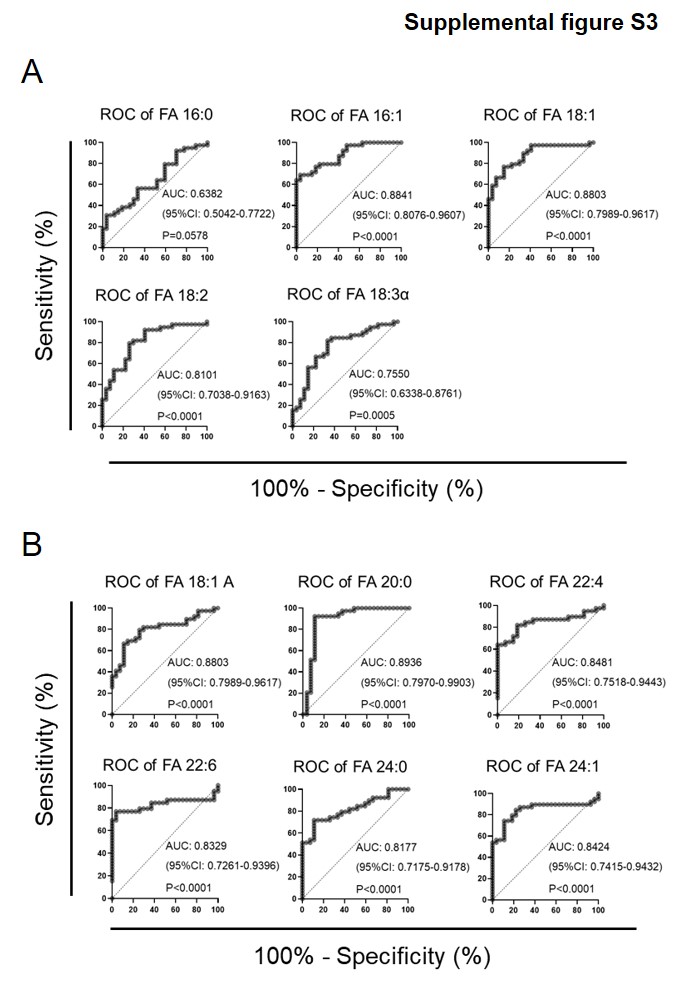


**Supplemental figure S3. Diagnostic potential of levels of 11 free fatty acids in patients with all stages of ovarian cancer.** ROC analysis of serum free fatty acids with increased **(A)** and decreased **(B)** values in patients with all stages of ovarian cancer compared with healthy controls.
