# Supplementary material for: Serum Free Fatty Acid Changes Caused by High Expression of Stearoyl-CoA Desaturase 1 in Tumor Tissues Are Early Diagnostic Markers for Ovarian Cancer
Source: Cancer Res Commun. 2023 Sep 13;3(9):1840–52. doi: 10.1158/2767-9764.CRC-23-0138 (PMC10498943; doi:10.1158/2767-9764.CRC-23-0138)
Supplement: Figure S4 — Supplemental figure S4. Serum free fatty acid levels in stage I and II ovarian cancer patients among histologic types, related to Figure 5. Comparison of serum free fatty acid levels in patients with stage I/II ovarian cancer patients of various histologic types (clear cell (n=10), serous (n=7), endometrioid (n=3), mucinous (n=6), others (n=4)). n.s., not significant. [file crc-23-0138-s04.docx]

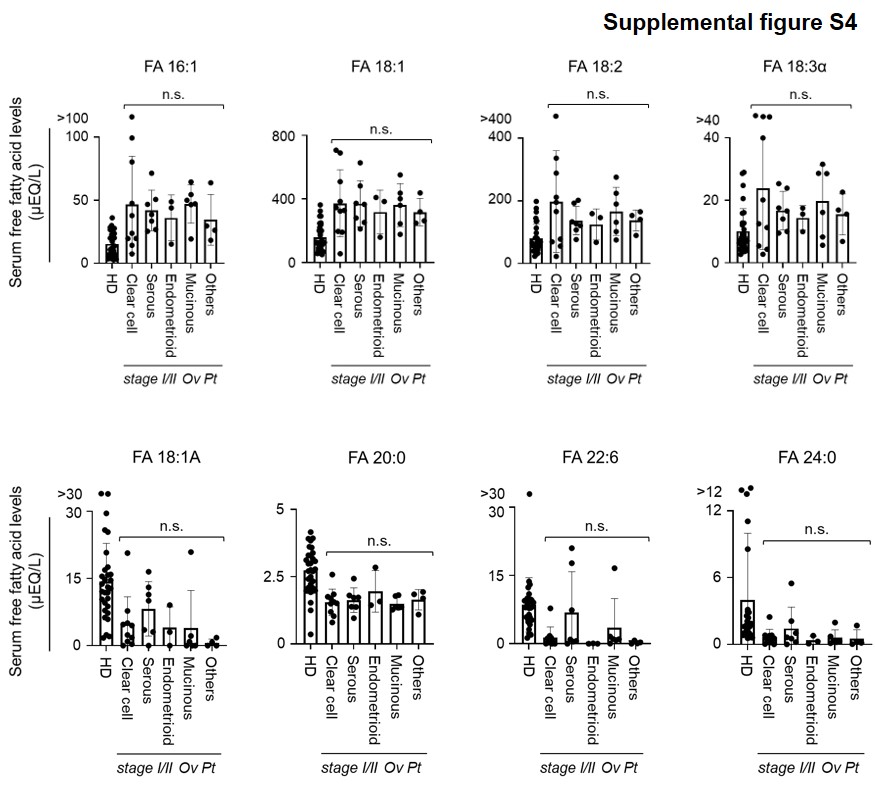


**Supplemental figure S4. Serum free fatty acid levels in stage I and II ovarian cancer patients among histologic types, related to Figure 5.** Comparison of serum free fatty acid levels in patients with stage I/II ovarian cancer patients of various histologic types (clear cell (n=10), serous (n=7), endometrioid (n=3), mucinous (n=6), others (n=4)). n.s., not significant.
