# Supplementary material for: Serum Free Fatty Acid Changes Caused by High Expression of Stearoyl-CoA Desaturase 1 in Tumor Tissues Are Early Diagnostic Markers for Ovarian Cancer
Source: Cancer Res Commun. 2023 Sep 13;3(9):1840–52. doi: 10.1158/2767-9764.CRC-23-0138 (PMC10498943; doi:10.1158/2767-9764.CRC-23-0138)
Supplement: Figure S5 — Supplemental figure S5. Correlation between serum free fatty acid levels and BMI in patients with early-stage ovarian cancer. Analysis of the correlation between serum free fatty acid levels and BMI with the ability to diagnose stage I and II ovarian cancer patients, shown in Figure 5A and B. [file crc-23-0138-s05.docx]

**
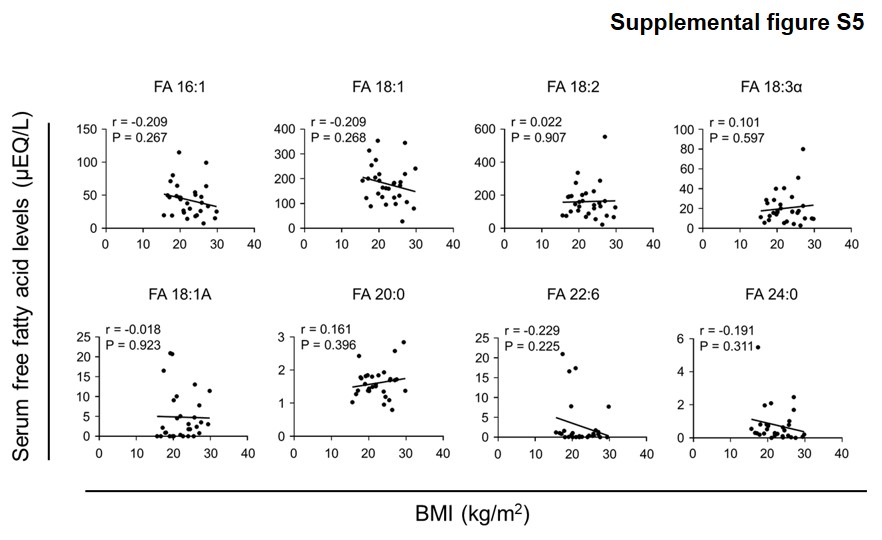
**

**Supplemental figure S5. Correlation between serum free fatty acid levels and BMI in patients with early-stage ovarian cancer.** Analysis of the correlation between serum free fatty acid levels and BMI with the ability to diagnose stage I and II ovarian cancer patients, shown in Figure 5A and B.
