# Supplementary material for: Serum Free Fatty Acid Changes Caused by High Expression of Stearoyl-CoA Desaturase 1 in Tumor Tissues Are Early Diagnostic Markers for Ovarian Cancer
Source: Cancer Res Commun. 2023 Sep 13;3(9):1840–52. doi: 10.1158/2767-9764.CRC-23-0138 (PMC10498943; doi:10.1158/2767-9764.CRC-23-0138)
Supplement: Figure S6 — Supplemental figure S6. Effect of short-term diet on serum free fatty acid levels. Serum was collected from healthy donors (n=7) pre and 2 hours post diet, and free fatty acids were measured. Gray lines represent individual values and black lines represent average values. n.s., not significant. [file crc-23-0138-s06.docx]

**
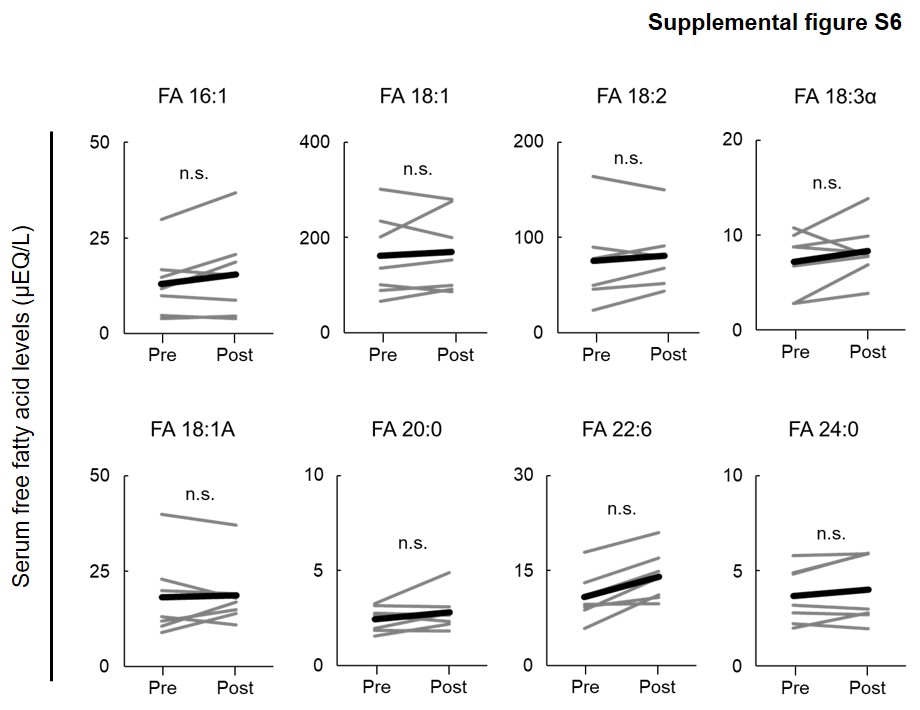
**

**Supplemental figure S6. Effect of short-term diet on serum free fatty acid levels.** Serum was collected from healthy donors (n=7) pre and 2 hours post diet, and free fatty acids were measured. Gray lines represent individual values and black lines represent average values. n.s., not significant.
