# Supplementary material for: Serum Free Fatty Acid Changes Caused by High Expression of Stearoyl-CoA Desaturase 1 in Tumor Tissues Are Early Diagnostic Markers for Ovarian Cancer
Source: Cancer Res Commun. 2023 Sep 13;3(9):1840–52. doi: 10.1158/2767-9764.CRC-23-0138 (PMC10498943; doi:10.1158/2767-9764.CRC-23-0138)
Supplement: Table S1 — Supplemental table S1. Characteristics of participants in the discovery set and the validation set. [file crc-23-0138-s07.docx]

**Supplemental table S1.** Characteristics of participants in the discovery set and the validation set.

|  |  | **Discovery Set** | | **Validation Set** | |
| --- | --- | --- | --- | --- | --- |
|  |  | healthy donors (n=21) | stage I/II OV ca Pt (n=20) | healthy donors (n=9) | stage I/II OV ca Pt (n=10) |
| **Age** | |  |  |  |  |
|  | median (range) | 40.7 (28-65) | 54.2 (33-80) | 39.6 (29-65) | 53.1 (38-74) |
|  |  |  |  |  |  |
| **Tumor size, *mm*** | |  |  |  |  |
|  | median (range) |  | 111.6 (30-280) |  | 109.0 (35-250) |
| **Histopathological subtypes, *n (%)*** | |  |  |  |  |
|  | Clear cell |  | 7 (35.0 %) |  | 3 (30.0 %) |
|  | Endometrioid |  | 2 (10.0 %) |  | 1 (10.0 %) |
|  | Serous |  | 5 (25.0 %) |  | 2 (20.0 %) |
|  | Mucinous |  | 4 (20.0 %) |  | 2 (20.0 %) |
|  | Others |  | 2 (10.0 %) |  | 2 (20.0 %) |
| **Pathological stage, *n (%)*** | |  |  |  |  |
|  | I |  | 15 (75.0 %) |  | 7 (70.0 %) |
|  | II |  | 5 (25.0 %) |  | 3 (30.0 %) |
| **Ascites** | |  |  |  |  |
|  | none/small/massive |  | 17/2/1 |  | 7/1/2 |
