# Supplementary material for: Serum Free Fatty Acid Changes Caused by High Expression of Stearoyl-CoA Desaturase 1 in Tumor Tissues Are Early Diagnostic Markers for Ovarian Cancer
Source: Cancer Res Commun. 2023 Sep 13;3(9):1840–52. doi: 10.1158/2767-9764.CRC-23-0138 (PMC10498943; doi:10.1158/2767-9764.CRC-23-0138)
Supplement: Table S2 — Supplemental table S2. Characteristics of DI (FA18:1, FA20:0) and DI (CA125) in the discovery set and validation set. Threshold was set by calculating Youden’s index by ROC analysis using the discovery set. DI (FA18:1, FA20:0): -0.7531, DI (CA125): -0.018. [file crc-23-0138-s08.docx]

**Supplemental table S2.** Characteristics of DI (FA18:1, FA20:0) and DI (CA125) in the discovery set and validation set. Threshold was set by calculating Youden’s index by ROC analysis using the discovery set. DI (FA18:1, FA20:0): -0.7531, DI (CA125): -0.018.

|  | **Discovery set** | | **Validation set** | |
| --- | --- | --- | --- | --- |
|  | Diagnostic index (FA18:1, FA20:0) | Diagnostic index (CA125) | Diagnostic index (FA18:1, FA20:0) | Diagnostic index (CA125) |
| Sensitivity (%) | 95.0 | 65.0 | 90.0 | 70.0 |
| Specificity (%) | 95.2 | 85.7 | 100.0 | 100.0 |
| False negative rate (%) | 5.0 | 35.0 | 10.0 | 30.0 |
| False positive rate (%) | 4.8 | 14.3 | 0.0 | 0.0 |
| Positive predictive value (%) | 95.0 | 81.3 | 100.0 | 100.0 |
| Negative predictive value (%) | 95.2 | 72.0 | 90.0 | 75.0 |
| Prevalence (%) | 48.8 | 48.8 | 52.6 | 52.6 |
